# 577-nm high-power optically pumped semiconductor laser is safe and effective in the treatment of inflammatory acne: a prospective, single-center, split-face comparative study

**DOI:** 10.1186/s40001-021-00573-z

**Published:** 2021-09-09

**Authors:** E. M. Mohamed, K. M. Tawfik, I. B. Elsayed, E. Bölke, P. A. Gerber

**Affiliations:** 1 Department of Dermatology, Al-Azhar University, Assiut, Egypt; 2 Department of Radiation Oncology, Medical Faculty, Heinrich Heine University of Duesseldorf, Duesseldorf, Germany; 3 Dermatology and Laser Center, Dermatologie Am Luegplatz, Duesseldorf, Germany; 4 Department of Dermatology, Medical Faculty, Heinrich Heine University of Duesseldorf, Duesseldorf, Germany

**Keywords:** 577-nm diode laser, Acne, Vascular laser

## Abstract

**Objective:**

This study aimed to appraise the efficacy of a 577-nm high-power optically pumped semiconductor laser (HOPSL) for the treatment of inflammatory acne.

**Methods:**

The study included 50 patients with acne vulgaris (inflammatory type), 14 men, and 36 women; patient ages ranged from 16 to 35 years. The left side of the face was treated with a single pass of a 577-nm high-power optically pumped semiconductor laser (HOPSL) every 2 weeks for 3 sessions. The severity of acne examined prior to the first session and 4 weeks after the last session (Investigator's Global Assessment of acne severity, IGA; single lesion count).

**Results:**

At baseline, no statistically significant difference in the severity of inflammatory acne lesions between both sides was observed. One month after the final session, a significant improvement (IGA reduction of > 50%) of the overall severity of acne was observed in 49 patients (98%) on the laser-treated side versus 41 (82%) the control side of the face (*P* < .05). Hence, we found a significant reduction in the mean percentage of inflammatory papules, pustules, and nodules on the laser-treated versus the control side (79.33 vs 56.92, 78.04 vs 43.33, 64.85 vs 21.93%, respectively) (*P* < 0.05). Side effects in the form of erythema and irritation during sessions were transient and tolerated by the patients.

**Conclusion:**

The 577-nm high-power optically pumped semiconductor laser is effective and safe for the treatment of inflammatory lesions (papules, pustules, and nodules) in acne patients.

## Introduction

Acne vulgaris is one of the most common skin conditions. Acne can be classified into non-inflammatory type (comedones), or inflammatory type (papules, pustules, and nodules) [1]. Acne commonly affects adolescents and young adults, can cause scarring, and can result in low self-esteem and affect mental health [2].

The pathogenesis of acne is multi-factorial and includes an overproduction of sebum, follicular hyper-keratinization, a colonization with Cutibacterium acnes, and a consecutive inflammation [3].

Available treatment options for acne include mainly topical and oral drugs. One of the problems of the topical treatment is that it requires frequent application (compliance), while the use of oral medications may be associated with more severe side effects [4]. It is particularly important to treat acne before scars begin to appear, as even the most up-to-date laser treatments cannot guarantee their full resolution [5].

Several types of lasers have been used to treat acne vulgaris in the past years. Of these, vascular lasers are reported to improve inflammatory acne lesions safely and efficiently [6]. Lasers are proposed to decrease Cutibacterium acnes and to reduce the pilosebaceous unit size and function [7]. Here, we aimed to assess the efficacy of a novel 577-nm high-power optically pumped semiconductor laser (HOPSL) in the treatment of inflammatory type acne.

## Patients and methods

We conducted a single-center, prospective, half-side controlled, split-face study approved by the Ethics Committee of Al-Azhar university hospital.

Exclusion criteria included non-inflammatory type acne, pregnant or lactating women, active herpes simplex infections, keloids or hypertrophic scars, photosensitivity, immuno-compromised patients, and patients who had received systemic or topical antibiotics in the last month or oral isotretinoin in the last 6 months.

A total of 50 patients (14 males and 36 females) with inflammatory acne, with a mean age of 21.62 years (range: 16–29 years) and a mean duration of acne of 3.5 years (range: 1–7 years) were included in the study. According to Fitzpatrick skin type, 14 patients (28%) were classified as type III, 32 patients (64%) were classified type IV and 4 patients (8%) were classified type V.

The left side of the face was treated with a single pass of a novel 577-nm high-power optically pumped semiconductor laser (HOPSL) (QuadroStarPRO, Asclepion Laser Technologies, Jena, Germany) for 3 sessions at 2-week intervals. Fluence was started with 17 J/cm^2^ in the first session and was increased by 2 J/cm^2^ in every added session; pulse duration ranged from 28 32 ms according to the skin photo-type; the laser was applied in scanner-mode with a coverage of 80%. Patients were advised to avoid sun exposure and use topical sun protection with SPF > 30. No added acne-specific treatments were performed during the study period.

Patients were evaluated at baseline and 4 weeks after the final laser-session by clinical examination and standardized photography (Canon PowerShot A3400 IS 16MP digital camera). Acne severity was quantified according to the Investigator's Global Assessment of Acne Severity Scale (IGA) and by single lesions count (inflammatory papules, pustules, and nodules).

## Statistical analysis

The statistical analysis was carried out using SPSS (Statistical Package for Social Sciences), version 21 (SPSS Inc. Chicago, IL, USA). Qualitative variables were expressed as frequency and percentage. Data were presented as mean ± standard deviation (SD) and the differences were evaluated by an independent sample *t*-test. A value of 0.05 or less was considered significant.

## Results

**Fig. 1.**
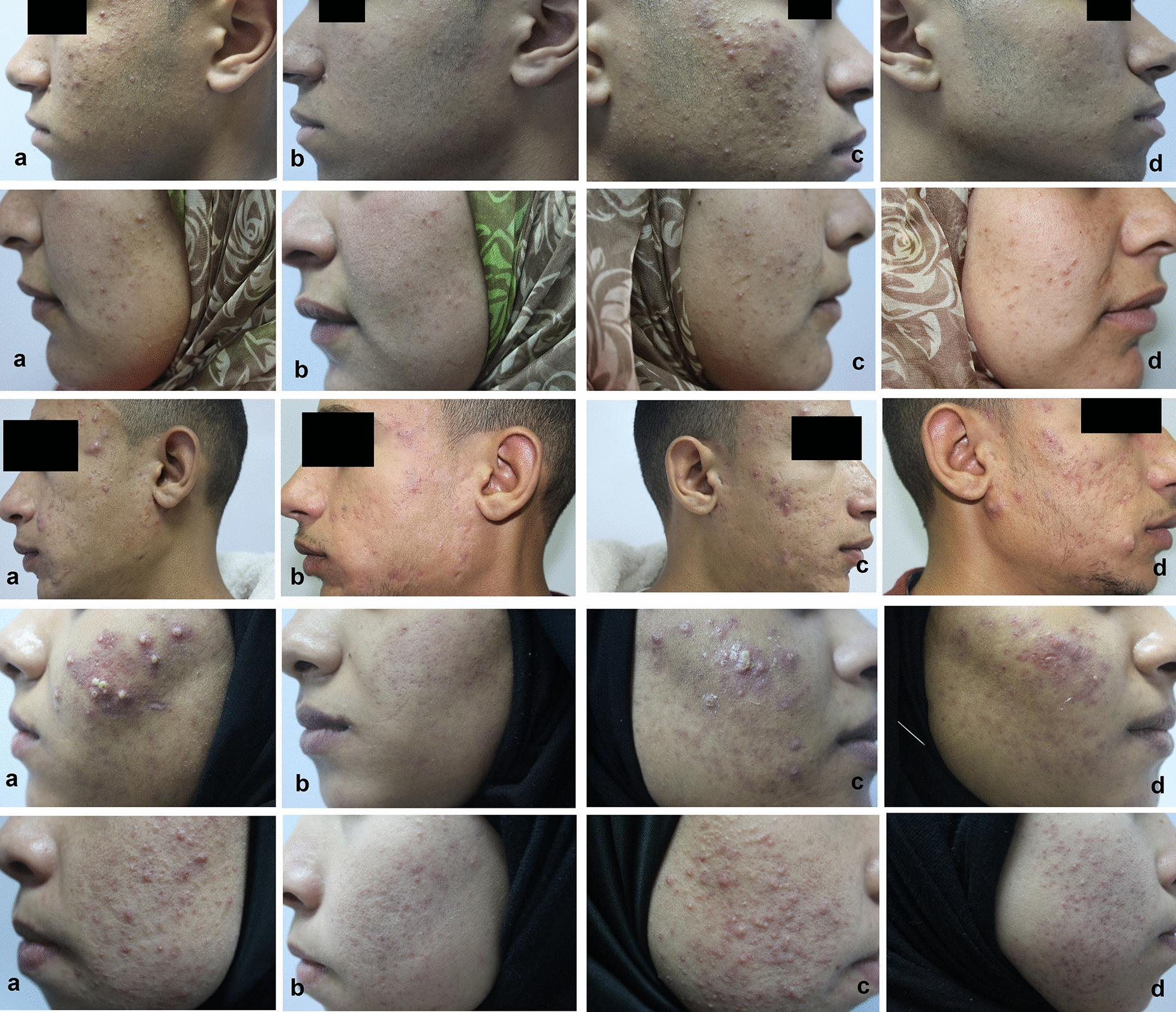
577-nm high-power optically pumped semiconductor laser (HOPSL) is effective in the treatment of inflammatory acne vulgaris: five representative cases, showing acne severity at baseline (**a**, **c**) and 4 weeks after treating the left side of the face with 3 sessions of 577-nm high-power optically pumped semiconductor laser (**b**) versus no treatment for the right side of the face (**d**). Clinical improvements were significantly better on the laser-treated side (**b** vs. **d**)

At baseline no statistically significant difference in acne severity was noted between laser and non-laser-treated sides. At the end of the study, a significant improvement in acne severity (IGA reduction > 50%) was seen in 49 patients (98%) on laser-treated side versus 41 (82%) on the non-laser-treated side of the face (*P* < 0.05) (Fig. 1; Table 1). At the final visit, there was a significant reduction in the mean percentage of inflammatory acne lesions at the laser-treated side vs. the non-laser-treated side (*P* < 0.05). In detail, we found a relative reduction of inflammatory papules (79.33 vs 56.92%), pustules (78.04 vs 43.33%), and nodules (64.85 vs 21.93%) for the laser-treated side vs. the non-laser-treated side (Fig. 1; Table 2). Reported side effects for the laser-treated side were mild and included transient erythema and irritation during sessions. Side effects were well-tolerated by the patients.

**Table 1:** Outcome of treatment (severity of acne, IGA)

	Laser-treated side	Non-laser-treated side	*p*-value
	*n* (%)	*n* (%)	
Clear (100%)	15 (30%)	8 (16%)	0.002*
Almost clear (75%–< 100%)	29 (58%)	11 (22%)	
Marked improvement (50–< 75%)	5 (10%)	22 (44%)	
Moderate improvement (25–< 50%)	0 (0%)	7 (14%)	
Slight improvement (1–< 25%)	0 (0%)	1 (2%)	
No change (0%)	1 (2%)	1 (2%)	

Chi-square test was used

Data expressed as *n* (%)

*P*. value < 0.05 is significant

**Table 2:** Mean percentage of improvement acne lesions after treatments

	Laser-treated side	Non-laser-treated side	*p*-value
Papule	79.33 ± 27.94 (0–100)	56.92 ± 27.20 (0–100)	0.001*
Pustule	78.04 ± 47.47 (0–100)	43.33 ± 47.49 (0–100)	0.001*
Nodule	64.85 ± 41.48 (0–100)	21.93 ± 38.88 (0–100)	0.001*

Independent *T* test was used

Data expressed as mean ± SD (range)

*P*. value < 0.05 is significant

## Discussion

Acne vulgaris is common skin diseases, which varies in severity between patients and may affect the psychological state of affected patients [8]. Acne can be classified into inflammatory and the non-inflammatory type. There are several ways to treat acne, including topical formulations and oral medications, as well as energy-based treatments. The treatment of choice or combinations thereof depends on disease type and severity, its effect on patients' psychological status, and the presence of contraindications to any line of treatment [9].

Laser- and intense pulsed light (IPL) systems have been have been proven as effective in the treatment of inflammatory acne with favorable side effects [10, 11]. Vascular lasers, including pulsed dye lasers (PDL), are one of the most common type of lasers used in acne treatment [6, 12]. Alexiades-Armenakas reported that 19 patients with inflammatory acne vulgaris achieved excellent responses after 595-nm PDL therapy [13]. Further studies confirmed significant effects for PDL therapy in acne patients [14], whereas another representative split-face study on 40 patients with facial acne treated with non-purpuric PDL did not show significant improvements [15].

Comparable to a 595-nm PDL, a novel 577-nm high-power optically pumped semiconductor laser (HOPSL) (QuadroStarPRO, Asclepion Laser Technologies, Jena, Germany) emits yellow light of a comparable wavelengths. HOPSL was shown to effectively treat various vascular and pigmented skin conditions [16–18]; yet, to date no study has assessed its efficacy in inflammatory acne.

In the present study, a single pass of the 577-nm HOPSL was applied to one half of the face of patients suffering from inflammatory acne, achieving a significant improvement in the vast majority of cases (significant improvements in IGA as well as single lesion count). Side effects were mild and well-tolerated.

In line with other vascular laser- and IPL-systems, the following mechanisms of action can be postulated: the 577-nm diode laser reduces Cutibacterium acnes through absorption of light by bacterial porphyrins and consecutive generation of reactive oxygen (ROS). Moreover, the laser generates photothermal effects via heating of the blood vessels and sebaceous glands [6, 19]. Finally, vascular laser treatments have been shown to induce transforming growth factor beta (TGFbeta), suggesting additional anti-inflammatory effects [9].

In conclusion, our study shows that the 577-nm high-power optically pumped semiconductor laser (HOPSL) is safe and effective in the treatment of inflammatory acne vulgaris.

## Data Availability

All data and materials can be accessed via MEEM and PAG.
